# Characteristics of Self-Rated Oral Health among Syrian Refugee Parents in Ontario

**DOI:** 10.1155/2023/4136520

**Published:** 2023-11-24

**Authors:** Aseel Alzaghoul, Parmin Rahimpoor-Marnani, Khalid Yunis, Akm Alamgir, Baraa Alghalyini, Hala Tamim

**Affiliations:** ^1^Division of Social and Transcultural Psychiatry, McGill University, Montreal, PQ, Canada H3A 1A2; ^2^Faculty of Health, York University, 4700 Keele Street, Toronto, ON, Canada M3J 1P3; ^3^Department of Pediatrics and Adolescent Medicine, American University of Beirut, Beirut, Lebanon; ^4^Access Alliance Multicultural Health and Community Services, 340 College Street, Suite 500, Toronto, ON, Canada M5T 3A9; ^5^Family Medicine, Alfaisal University, Riyadh 50927, Saudi Arabia; ^6^School of Kinesiology and Health Science, York University, 4700 Keele Street, Toronto, ON, Canada M3J 1P3; ^7^College of Medicine, Alfaisal University, Riyadh 50927, Saudi Arabia

## Abstract

**Background:**

Canada has been hosting Syrian refugees since early 2015. Almost half of the Syrian refugee population lives in Ontario, with dental health being at the top of the list of important immediate needs. The objective of the study was to evaluate self-rated oral health and its associated factors among Syrian refugee parents residing in Ontario.

**Methods:**

This was a cross-sectional study where 540 Syrian refugee parents, residing in Ontario and with at least one child less than 18 years of age, were interviewed. Information about self-rated oral health was collected based on the question “In general, how would you rate the health of your teeth and mouth?” with answers ranging from 1 representing “excellent” and 5 representing “very poor.” Multiple linear regression analysis was performed to assess the independent relationship between each of the sociodemographic-, migration-, health-, dental-related factors, and self-rated oral health.

**Results:**

The overall prevalence of poor and very poor self-rated oral health was 43.5%. The results showed that the presence of dental health insurance, private sponsorship, improved physical and mental health, and regular visits to the dentist were factors related to improved oral health. *Discussion*. To achieve better oral health outcomes among refugee populations, including Syrian refugees, efforts should be focused on improving dental care and dental insurance for vulnerable populations.

## 1. Background

Since the civil war started in Syria in 2011, more than 250 thousand Syrian civilians have been killed, and 22 million have been displaced [1]. Many countries responded to this crisis by hosting refugees; Canada alone hosted 44,000 Syrian refugees between early 2015 and mid-2021 [2]. The Canadian province of Ontario hosted almost half of the Syrian refugee population, reaching 19,865 in June 2021 [2]. As the number of newcomers increases every year, health needs increase as well; among the most important and immediate needs, dental health is on top of the list, as studies have found [1, 3]. As part of the settlement for Syrian refugees in Canada, they become immediately eligible for provincial health coverage [1]; however, such coverage does not include oral health or other specialized health care. Therefore, the Canadian government initiated the Interim Federal Health Program (IFHP) to cover services such as mental health and dental care for all refugees [1, 4–7]. However, the drawbacks of this program are that it is temporary for 12 months [4, 6], limited for emergency and basic dental care [7], and a person must be recognized by the federal government as a refugee before entering Canada to be eligible for the program [5]. As a result of the limitations of such programs, refugees and asylum seekers from different origins reported negative dental care experiences, such as delayed consultation, limited care and treatment choices, and long wait times [8].

The Canadian Dental Association recommends that individuals visit the dentist at least once every 6 months [9, 10], as regular dental visits are not only to prevent oral health deterioration but also to prevent further risky medical complications such as cardiovascular complications [11, 12]. However, a study on immigrants in Ontario—the province that has one-third of the immigrant population in Canada—showed that visiting the dentist only for emergencies was reported by 25% of immigrants [12] compared to the 19.3% reported by the general Canadian population [12]. Studies have shown that adult refugees' and immigrants' oral health deteriorates due to their irregular visits for dental care in their first years in Canada [8, 11]. Refugees face postmigration challenges, such as social discrimination and isolation, lack of economic opportunity, lack of access to resources, and poor language proficiency [13, 14]. A study showed that the percent of immigrants reporting a dental problem tripled 2 years after immigration: 0–6 months (9.4%, 95% confidence interval (CI): 8.6%–10.3%) and 6 months to 2 years (32.6%; 95% CI: 31.6%–33.5%) [11]. Additionally, dental health status, especially in regard to conditions such as untreated decay, among refugees has been shown to be at poorer status compared to other minority populations, such as Indigenous populations [15].

Perceived oral health has been associated with insurance coverage and several sociodemographic-, economic-, and health-related factors [7, 8, 10, 12, 16]. With regard to refugee populations, some barriers to accessing oral health have also been identified, including general health literacy, language, and policy barriers [7, 8]. Little is currently known about the oral health status and frequency of dentist visits among Syrian refugees in Canada. It is essential to explore such outcomes in vulnerable populations to ensure the overall health and well-being of our communities and to enhance provincial and country policies for Syrian refugees' access to dental health [8]. The main purpose of this study was to describe self-rated oral health among Syrian refugees in Ontario and to assess its relationship with several sociodemographic-, migration-, health-, and oral health-related factors.

## 2. Methods

This is a cross-sectional study with a total of 540 Syrian refugee parents who were recruited and interviewed between March 2021 and March 2022. The inclusion criteria required the participants to be Syrian refugee parents residing in Ontario, Canada, have at least one child under the age of 18 years at the time of the interview, and have resettled in Canada after 2015. Power analysis showed that for multiple linear regression analysis, with 18 independent variables, an *α* of 0.05 and a medium effect size of 0.15, a minimum sample size of 183 is required to achieve a power of 90% [17]. Participants were recruited and interviewed through telephone interviews to comply with social distancing guidelines considering the COVID-19 pandemic [18]. The participants were recruited through convenience sampling with the help of organizations, including Access Alliance Multicultural Health and the Arab Community Centre of Toronto. Research assistants who could read, write, and speak in Arabic, specifically in the Syrian dialect, administered the survey. Informed consent was obtained from all participants prior to participating in the study. To comply with social distancing recommendations due to the COVID-19 pandemic, questionnaire surveys were conducted remotely over the phone. Before the administration of the questionnaire, the research assistant sent electronically to participants a soft copy of the consent form. The research assistant then went over the consent form and answered any questions the participants had. The research assistant then recorded the audio of the participants' oral consent. Participants received a $20 honorarium for their participation in the study. The project was approved by the Research Ethics Board at York University (Certificate #e2019-128).

Information about self-rated oral health, the main dependent variable, was collected based on the question “In general, how would you rate the health of your teeth and mouth?” with answers ranging from 1 representing “excellent” and 5 representing “very poor.” Several sociodemographic-, migration-, health-, and dental-related factors were also collected from all participants. The sociodemographic characteristics considered for the study included gender roles (being a mother/father), age, number of children, highest level of education (none/elementary/secondary high-school diploma/university), and employment status (yes/no). In addition, information on the perceived level of Canadian languages—English or French—was also collected on a 6-point Likert scale, with 1 representing “excellent” and 5 representing “not at all.” The migration-related factors included having resided previously in a refugee camp (yes/no), type of sponsorship involved in the process of becoming a refugee (government-assisted refugee (GAR)/privately sponsored refugee (PSR)/blended/other), and number of years spent in Canada. Health-related factors, including information on self-rated physical and mental health, were collected on a 5-point Likert scale, with 1 representing an “excellent” rating and 5 representing a “poor” rating, alcohol consumption (yes/no), and smoking. Information on dental health-related factors included whether participants had access to oral care insurance (full coverage/partial coverage/no coverage) and whether they visited the dentist only for emergencies.

Simple linear regression models were performed to assess the bivariate relationship between each of the sociodemographic-, migration-, health-, dental-related factors, and self-rated oral health. In addition, one multiple linear regression model was conducted with the dependent variable being self-rated oral health and the independent variables being all the sociodemographic-, migration-, health-, and dental-related factors. The beta coefficient and 95% CIs were reported. All regression models were adjusted for the clustering effect of belonging to the same family. All analyses were conducted using the Statistical Package for the Social Sciences (SPSS, version 26.0).

## 3. Results

The total sample included in the present study was 540 Syrian refugee parents in Ontario. The average age of participants was 39.7 years (SD = 7.3) and they had 3.35 children on average (SD = 1.4), respectively, with 60.9% of participants being mothers. The percentage of participants who had a high school level of education or higher was 81.3%; however, 65.6% of participants were unemployed. The average amount of years spent in Canada from the date of arrival to the interview date was 4.28 (SD = 1.6). Of all participants, only 6.3% rated their oral health as excellent, 26.9% as good, 23.1% as fair, 24.8% as poor, and 18.7% as very poor. Only 4.6% of participants had complete oral health insurance coverage. Additionally, 72.8% of participants visited a dentist only for emergencies (Table 1).

The multiple linear regression model had an overall *R* square of 0.211 (Table 2). Although the sociodemographic factors, number of children, and work status were significant at an *α* of 0.05 at the bivariate analysis level, both variables lost their significance after performing multiple linear regression analysis and adjusting for the other variables. However, the variables age and education approached significance for the multiple linear regression analysis wherein older participants had worse self-rated oral health (Adj*β* = 0.01; *p*=0.071), and participants with university-level education had improved self-rated oral health compared to participants with no education or elementary education (Adj*β* = −0.33; *p*=0.088). Furthermore, the results of the multiple linear regression model showed that participants who were privately sponsored had improved self-rated oral health compared to participants who were sponsored by the government (Adj*β* = −0.29; *p*=0.025). Additionally, participants with higher self-rated physical health (Adj*β* = 0.16; *p*=0.002) and mental health (Adj*β* = 0.09; *p*=0.044) had improved self-rated oral health. The results of the multiple linear regression model showed that participants who had access to oral care insurance, whether it provided full coverage (Adj*β* = −0.83; *p*=0.001) or partial coverage (Adj*β* = −0.24; *p*=0.028), exhibited significantly improved self-rated oral health in comparison to those without insurance. Finally, participants who visited dentists only for emergencies had worse self-rated oral health than other participants (Adj*β* = 0.28; *p*=0.018).

## 4. Discussion

This study aimed to evaluate the self-rated oral health and factors associated with this among Syrian refugee parents residing in Ontario. The results from this study highlighted that 43.5% of participants rated their oral health as poor or very poor, and 56.9% of the participants had no dental health insurance. In addition, the findings indicated that the presence of partial or complete insurance contributed significantly to improved self-rated oral health. Other factors, such as private sponsorship type, improved physical and mental health, and regular visits to the dentist, were also related to self-rated improved oral health. Overall, these findings are valuable, as they can assist in guiding the government to improve refugees' support programs to include dental insurance coverage, which will help with enhancing the perceived quality of oral health and can be seen as a strategy to enhance the utilization of dental care among Syrian refugees.

Compared to previous studies [12, 16, 17] using the same question of self-rated oral health, this study showed that Syrian refugees had higher poor/very poor self-rated oral health of 43.5% compared to Canadians' self-rated oral health in which 5% [10] and 11.5% [19] of studies participants reported poor self-rated oral health, and 7.5% [10, 19, 20] of participants reported fair to poor self-rated oral health. The data of this study also show a higher prevalence of poor/very poor self-rated oral health compared to studies focused on the Indigenous population's self-rated oral health in Canada with a poor rating prevalence of 18.5% [19]. In the United States, a group of war-affected refugees living in Texas reported a similar percentage of poor/very poor self-rated oral health of 47.3% in comparison with Syrian refugees in Canada [21]. Among the sociodemographic factors, age and education were significantly associated with self-rated oral health. Older age was associated with poor self-rated oral health, consistent with findings of a previous study in Ontario, where among various age groups (immigrants and Canadians), those older than 18 years, were at increased risk of poor oral health [10, 12]. This could be related to the nature of oral problems that tend to accumulate with time, resulting in oral diseases with the advancement of age [22]. Similar to this study, previous studies also showed that those who lack high school education were significantly more likely to report poor oral health [10, 23]. This result has been linked to the association between low education and low income, which prevents utilization of services, thus causing poor oral health [10, 24].

One finding of this study was that PSRs had a significantly better perception of their oral health (*p* < 0.001) than their governmentally sponsored counterparts (GARs) or refugees that were sponsored through other means. A follow-up study on Canadian refugees also showed that GARs from Syria are at significantly higher odds of reporting unmet health care needs, most commonly dental care, than PSRs when the type of sponsorship is the only variable being analyzed [25]. Despite the lack of full financial coverage for dental care through PSR and GAR, sociodemographic and other differences exist between refugees that are sponsored through these two program types. GARs are typically assigned to governmentally funded Service Provider Organizations (SPOs) to receive financial support, while PSRs are supported by sponsoring groups of community sponsors. The latest has been shown to contribute to strengthening refugees' social networks when compared to GARs [25, 26]. Therefore, PSRs are more likely to feel connected to their communities and learn how to utilize health care services available to them to a greater extent [25], which can also contribute to a better self-rate of oral health. Of the health factors, our study found that poor physical and mental health were strongly associated with poor self-rated oral health. These findings align with studies among refugees in Jordan, where newly arrived refugees and those with lower health-related quality of life (HRQOL) scores had significantly higher oral disease levels than the general population due to postmigration stress [27].

Another primary finding was that access to insurance programs that provide complete coverage of dental care costs can help with significantly enhancing the perceived quality of oral health (*p* < 0.001) among Syrian refugees when compared to insurance programs with partial coverage of costs or no access to an insurance program, which was associated with the worst perception of oral health. Consistent with two studies by Bhusari et al. [28] and Barazanchi et al. [29], the lack of insurance coverage was significantly associated with a poor self-rate of oral health among this refugee population. Another study with Syrian refugee adults in San Antonio, Texas, showed that 41.5% of the participants reported that they did not have enough money to access dental care [20]. Therefore, these results highlight the significant role that insurance programs, especially those that provide full coverage of health care costs, can play in terms of ensuring that the oral care costs of Syrian refugees are met appropriately and in a timely manner.

Finally, in the current study, a greater likelihood of poor dental health self-rate was also observed among Syrian refugees who only visited the dentist for emergencies. This is consistent with a previous study [12] with immigrant populations in Ontario in which immigrants who rated their oral health as fair or poor were more likely to visit a dentist only for emergencies. A possible explanation would be related to the barriers that are preventing them from visiting a dentist regularly, such as their working hours, the discrimination they might face as refugees, social exclusion, and other social determinants of health. Additionally, previous studies found that refugees would seek treatment late in the course of oral health problems due to psychosocial factors, including dental anxiety, the accessibility and availability of dental services, as well as financial factors, and their perception of the importance of regular dental care [29]. Consequently, they would only seek emergency care upon the worsening of symptoms [12].

## 5. Conclusion

This study examined self-rated oral health and its association with sociodemographic-, migration-, health-, and dental health-related factors among a Syrian refugee population in Ontario. The estimated poor to very poor oral health self-rate was high, at 43.5%, and 72.8% of subjects reported visiting dentists for emergency purposes only. The presence of dental health insurance, private sponsorship, improved physical and mental health, and regular visits to the dentist were factors related to improved oral health. Furthermore, these findings highlight the need for efforts to improve dental care and dental insurance among refugee populations, including Syrian refugees, to achieve better oral health outcomes. The average years since arriving to Canada for study participants were 4.28 years; accordingly, long-term data are needed to confirm the findings of this study. Additionally, qualitative studies can provide information about participants' experiences and barriers to oral health services in Canada.

## Figures and Tables

**Table 1:** Characteristics of study participants.

Factors	Number	Percentage (%)	Mean (SD)
Sociodemographic characteristics			
Gender			
Mother	329	60.9	
Father	211	39.1	
Age			39.7 (7.32)
Number of children			3.35 (1.47)
Education			
None/elementary	101	18.7	
Secondary high-school diploma	278	51.1	
University	161	29.8	
Working status			
Yes	186	34.4	
No	354	65.6	
Canadian language self-rate ^*∗*^			3.03 (1.23)
Migration-related factors			
Lived in a refugee camp			
Yes	62	11.5	
No	472	87.4	
Sponsorship			
Governmental	202	37.4	
Private	312	57.8	
Blended and others	26	26	
Years spent in Canada			4.28 (1.66)
Health-related factors			
Physical health self-rate ^*∗*^ ^*∗*^			2.89 (1.08)
Alcohol drinking			
Yes	78	14.4	
No	462	85.6	
Smoking			
Cigarettes or narghile	237	43.9	
None	303	56.1	
Mental health self-rate ^*∗*^ ^*∗*^			3.06 (1.18)
Dental health-related factors			
Dental health insurance			
Complete coverage	25	4.6	
Partial coverage	206	38.1	
None	307	56.9	
Emergency visits			
Yes	393	72.8	
No	140	25.9	

^*∗*^Scale from 1 to 6 (1 = excellent and 6 = not at all);  ^*∗*^ ^*∗*^Scale from 1 to 5 (1 = excellent and 5 = poor).

**Table 2:** Results of the bivariate and multivariate analyses of association between sociodemographic characteristics, migration-related factors, health-related factors, oral health-related factors, and self-rated oral health.

Factor	Unadjusted *β* (SE)	95% CI	*p*-Value	Adjusted *β* (SE)	95% CI	*p*-Value
Sociodemographic factors						
Gender^a^						
Father	0.07 (0.10)	−0.13 to −0.28	0.469	−0.09 (0.12)	−0.33 to −0.15	0.468
Age	0.01 (0.00)	0.00–0.03	**0.011**	0.01 (0.00)	−0.00 to −0.03	0.071
Number of children	0.14 (0.03)	0.07–0.21	**<0.001**	0.00 (0.04)	−0.08 to −0.08	0.982
Education^b^						
Secondary high-school diploma	−0.34 (0.13)	−0.62, −0.07	**0.012**	−0.13 (0.15)	−0.43 to −0.17	0.389
University	−0.75 (0.15)	−1.06, −0.45	**<0.001**	−0.33 (0.19)	−0.71 to −0.05	0.088
Working status						
Yes	Ref			Ref		
No	−0.28 (0.11)	−0.50, −0.07	**0.009**	−0.00 (0.12)	−0.24 to −0.23	0.990
Canadian language self-rate ^*∗*^	0.22 (0.04)	0.14–0.30	**<0.001**	0.04 (0.05)	−0.06 to −0.15	0.450
Migration-related factors						
Lived in a refugee camp						
Yes	0.49 (0.16)	0.17–0.81	**0.003**	0.17 (0.16)	−0.14 to −0.49	0.280
No	Ref			Ref		
Sponsorship^c^						
Private	−0.52 (0.11)	−0.74, −0.30	**<0.001**	−0.29 (0.12)	−0.54, −0.03	**0.025**
Blended	−0.36 (0.24)	−0.85 to −0.12	0.139	−0.15 (0.24)	−0.63 to −0.31	0.513
Years spent in Canada	−0.06 (0.03)	−0.12 to −0.00	0.088	−0.04 (0.03)	−0.11 to −0.04	0.197
Health-related factors						
Physical health self-rate ^*∗*^ ^*∗*^	0.28 (0.04)	0.19–0.38	**<0.001**	0.16 (0.05)	0.06–0.27	**0.002**
Alcohol drinking						
Yes	−0.20 (0.15)	−0.49 to −0.09	0.182	0.03 (0.15)	−0.26 to −0.34	0.799
No	Ref			Ref		
Smoking						
Cigarettes or narghile	0.21 (0.10)	0.00–0.41	**0.043**	0.16 (0.10)	−0.04 to −0.37	0.118
None	Ref			Ref		
Mental health self-rate ^*∗*^ ^*∗*^	0.24 (0.04)	0.16–0.32	**<0.001**	0.09 (0.04)	0.00–0.19	**0.044**
Dental-health related factors						
Dental health insurance^d^						
Complete coverage	−1.01 (0.24)	−1.50, −0.053	**<0.001**	−0.83	−1.33 to −0.32	**0.001**
Partial coverage	−0.35 (0.11)	−0.57, −0.14	**0.001**	−0.24	−0.45, −0.02	**0.028**
Emergency visits						
Yes	0.58 (0.11)	0.35–0.81	**<0.001**	0.28	0.04–0.52	**0.018**
No	Ref			Ref		

^*∗*^Scale from 1 to 6 (1 = excellent and 6 = not at all),  ^*∗*^ ^*∗*^Scale from 1 to 5 (1 = excellent and 5 = poor). Reference category: ^a^mothers, ^b^none/elementary education, ^c^governmental sponsorship, and ^d^none (having no insurance). Bold values are significant at *α* = 0.05.

## Data Availability

The overall data are not freely available because the study involves collecting information from a vulnerable population, which raises identification concerns. However, those interested in accessing the data used or analyzed can make a reasonable request to the corresponding author.

## Additional Points

*Limitations*. Although this is the first study, to our knowledge, that investigates the oral health status among Syrian refugees in Ontario, several limitations must be noted. First, the cross-sectional design of the study may involve a reverse causality between self-rated oral health and the sociodemographic-, migration-, and health-related variables. Second, there is the possibility of selection bias as a result of voluntary participation in the study. Third, as all responses were collected by self-report, information bias may be present in addition to response bias that is highly related phenomenon in behavioral and health care research [30]. Self-reported data are prone to bias, as individuals tend to offer biased estimates of self-assessed behavior; this might range from a misunderstanding of a question or group of questions to social desirability bias, in which participants aim to a good image in the survey [30]. Finally, the results may also be subject to confounding biases, such as participants' oral health habits in Syria and before migrating to Canada, which were not collected for the present study.
